# The Antimicrobial Photoinactivation Effect on *Escherichia coli* through the Action of Inverted Cationic Porphyrin–Cyclodextrin Conjugates

**DOI:** 10.3390/microorganisms10040718

**Published:** 2022-03-26

**Authors:** Cláudia P. S. Ribeiro, Maria A. F. Faustino, Adelaide Almeida, Leandro M. O. Lourenço

**Affiliations:** 1 LAQV–REQUIMTE and Department of Chemistry, University of Aveiro, 3810-193 Aveiro, Portugal; claudia.santos.ribeiro7@ua.pt (C.P.S.R.); faustino@ua.pt (M.A.F.F.); 2 CESAM and Department of Biology, University of Aveiro, 3810-193 Aveiro, Portugal

**Keywords:** porphyrin, methoxypyridinium, thiopyridinium, Gram-negative bacterium, *E. coli*, planktonic form, cyclodextrins, positive charge

## Abstract

Photodynamic action has been used for diverse biomedical applications, such as treating a broad range of bacterial infections. Based on the combination of light, dioxygen, and photosensitizer (PS), the photodynamic inactivation (PDI) approach led to the formation of reactive oxygen species (ROS) and represented a non-invasive, non-toxic, repeatable procedure for pathogen photoinactivation. To this end, different tetrapyrrolic macrocycles, such as porphyrin (Por) dyes, have been used as PSs for PDI against microorganisms, mainly bacteria. Still, there is significant room for improvement, especially new PS molecules. Herein, unsymmetrical new pyridinone (**3**–**5**) and thiopyridyl Pors (**7**) were prepared with α-, β-, or γ-cyclodextrin (CD) units, following their quaternization to perform the corresponding free-base Pors (**3a**–**5a** and **7a**), and were compared with the already-known Pors **6a** and **8a**, both bearing thiopyridinium and CD units. These water-soluble porphyrins were evaluated as PSs, and their photophysical and photochemical properties and photodynamic effects on *E. coli* were assessed. The presence of one CD unit and three positive charges on the Por structure (**3a**–**5a** and **7a**) enhanced their aqueous solubility. The photoactivity of the cationic Pors **3a**–**5a** and **6a**–**8a** ensured their potential against the Gram-negative bacterium *E. coli*. Within each series of methoxypyridinium vs thiopyridinium dyes, the best PDI efficiency was achieved for **5a** with a bacterial viability reduction of 3.5 log_10_ (50 mW cm^−2^, 60 min of light irradiation) and for **8a** with a total bacterial viability reduction (>8 log_10_, 25 mW cm^−2^, 30 min of light irradiation). Here, the presence of the methoxypyridinium units is less effective against *E. coli* when compared with the thiopyridinium moieties. This study allows for the conclusion that the peripheral charge position, quaternized substituent type/CD unit, and affinity to the outer bacterial structures play an important role in the photoinactivation efficiency of *E. coli*, evidencing that these features should be further addressed in the pursuit for optimised PS for the antimicrobial PDI of pathogenic microorganisms.

## 1. Introduction

*Escherichia coli* belongs to the *Enterobacteriaceae* family, considered pathogenic Gram-negative bacteria. This bacterium is present in the intestinal microbiome of humans [1]. However, some strains can promote disease. Many of them cause severe infections in the urinary tract, digestive system, or even in the blood [2]. In recent decades, an increase in microbial resistance against antibiotics has been witnessed. *E. coli* is no exception and, thus, it is essential to find an alternative for its effective combat [3].

One of the interesting alternatives that have been studied, with promising results, is the antimicrobial photoinactivation (PDI) approach. PDI is based on the use of three key elements: the photosensitizer (PS), light source, and dioxygen (O_2_), which, when combined, promote the production of reactive oxygen species (ROS) and free radicals, inducing oxidative damage in biological targets [4,5]. This method can be applied in different microorganisms, such as bacteria [6], viruses [7], fungi [8], and protozoa [9]. Thus, the PDI approach has been explored for, e.g., surface disinfection [10], wastewater treatment [11], or food decontamination [12,13].

The selection of the PS is essential, which must be fast, efficient, and selective. Numerous molecules can be considered PSs, such as chlorin (Chl) [4,14,15,16], porphyrin (Por) [6,17,18,19], and phthalocyanine (Pc) dyes [5,20,21,22,23,24,25,26]. The PDI’s effectiveness is also influenced by the microorganism structure. The cell wall complexity of Gram-negative bacteria is higher than that of Gram-positive ones. However, their porous cell wall allows the crossing and accumulation of PS [21,27,28]. To improve the PS biodistribution, finding suitable PS chemical structures and solubility strategies are necessary. Some published studies demonstrated that cationic PSs have a higher efficacy against Gram-negative bacteria than neutral or anionic compounds [29,30]. The addition of positive charges on the Por periphery can also increase its photophysical properties and may result in an effective increase in the PDI treatment [31,32]. Moreover, binding porphyrin derivatives to amphiphilic molecules may be another strategy to adopt to increase their compatibility and biological efficacy [33,34,35]. Thus, the association between unsymmetrical porphyrins and versatile cyclodextrin (CD) units is extremely important for promoting water-soluble PS drugs [17]. This association with cyclic oligosaccharides derivatives, such as α-, β-, or γ-cyclodextrin (α-, β-, or γ-CD) units have been explored in drug design due to their water solubility, low toxicity, and low inflammatory response [36,37,38]. Moreover, CDs present several strategies for combating microbial infections, including reducing resistance to known antibiotics by altering the possibility of cell-to-cell communication (quorum sensing), thus reducing the likelihood of resistance [39,40,41]. Given these properties and those of porphyrins, it was decided to bring both together and try to increase the PDI efficacy against bacteria.

**Scheme 1:**
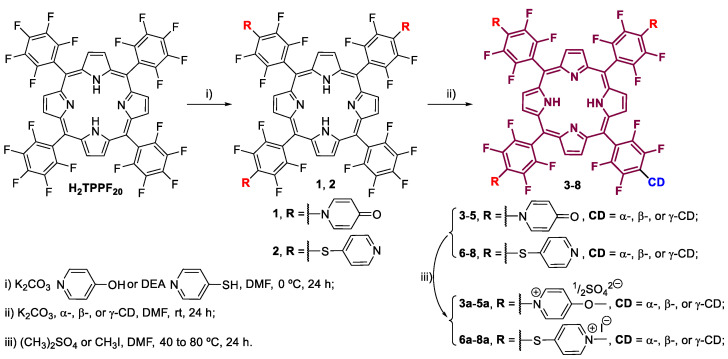
Cationic porphyrin-cyclodextrin conjugates.

Here, we synthesized water-soluble porphyrin–cyclodextrin (Por–CD) conjugates to assess their photodynamic efficiency against *E. coli*, a Gram-negative bacterial model. We compared the effectiveness of Por–CD conjugates (with three positive charges) bearing methoxypyridinium (**3a**–**5a**) and thiopyridinium (**6a**–**8a**) moieties to photoinactivate the *E. coli*. The new synthesized unsymmetrical Pors **3a**–**5a** and **7a**, covalently linked to the α-, β-, or γ-CD units, were compared with the already-known Pors **6a** and **8a** (Scheme 1).

## 2. Experimental Procedure

### 2.1. General

All reagents were acquired from Sigma-Aldrich (St. Louis, MO, USA) with high purities and were immediately used in the reactions. In some cases, standard procedures were applied on the solvents, such as distillation or drying [42]. Thin-layer chromatography (TLC) was performed in silica (Merck 60, 0.2 mm thick). Chromatography of the column was made in silica (Merck, Kenilworth, NJ, USA, 35–70 mesh). ^1^H (300.13 MHz) and ^19^F (282.38 MHz) NMR spectra were acquired in Bruker Avance-300 spectrometers. It was used as an internal reference of tetramethylsilane (TMS) and the deuterium solvent of dimethyl sulfoxide (DMSO-*d_6_*). Chemical shifts were determined in *δ* (ppm), and coupling constants (*J*) in Hertz (Hz). ESI–MS spectra were acquired on an instrument of Q-TOF 2 (Micromass, Manchester, UK). Sample solutions were prepared at 1 mg/mL in MeOH or H_2_O. The absorbance and steady-state fluorescence spectra of Pors (**3a**–**8a**) were recorded in dimethyl sulfoxide (DMSO) in quartz optical cells at 298 K and under normal air conditions by using Shimadzu UV–2501PC and the spectrofluorometer FluoroMax Plus, Horiba, respectively. The fluorescence quantum yield (Φ_F_) of the Pors was calculated by comparing the area under the emission spectrum of every compound, with the area under the emission spectrum of 5,10,15,20-tetraphenylporphyrin (**TPP**) (Φ_F_ = 0.13 in DMSO) as a standard reference [43].

### 2.2. Synthesis of 5-(pentafluorophenyl)-10,15,20-tris[2,3,5,6-tetrafluoro-4-(4-oxopyridin-1(4H)-yl)phenyl]porphyrin, Por ***1***

In a 50 mL round-bottom flask, 5,10,15,20-tetrakis(pentafluorophenyl)porphyrin (**H_2_TPPF_20_**, 300.3 mg, 3.1 × 10^−4^ mol, 1 eq.) and 4-hydroxpyridine (90.8 mg, 9.5 × 10^−4^ mol, 3.1 eq.) were dissolved in 10 mL of DMF under an N_2_ atmosphere. After stirring during the 48 h at r.t., the TLC analysis confirmed that the nucleophilic reaction was complete. The DMF was completely evaporated under reduced pressure. The crude reaction was dissolved in a mixture of CH_2_Cl_2_/MeOH (93/7, *v/v*%) and purified by column chromatography using the same eluent. The main fraction was subsequently precipitated in hexane, filtrated, and washed with the same solvent. The obtained purple solid was dried under a vacuum system over 6 h at 80 °C. Yield (Por **1**): 80% (297.6 mg, 3.3 × 10^−4^ mol).

^1^H NMR (300.13 MHz, DMSO–*d*_6_): *δ* −3.14 (s, 2H, -NH), 6.55 (d, *J* = 7.7 Hz, 6H, *m*-H), 8.23 (d, *J* = 7.7 Hz, 6H, *o*-H), 9.45–9.50 (m, 8H, β-H) ppm. ^19^F NMR (282.38 MHz, DMSO–*d*_6_): *δ* −158.67–−158.48 (m, 2F, Ar-F), −149.75 (t, *J* = 22.6 Hz, 1F, Ar-F), −145.42–−145.21 (m, 6F, Ar-F), −135.32 (dd, *J* = 26.0, 7.6 Hz, 2F, Ar-F), −135.07 –−134.88 (m, 6F, Ar-F) ppm. ESI–MS *m/z*: 1200.4 [M+H]^+^ and 601.0 [M+2H]^2+^.

### 2.3. Synthesis of Por–CD Conjugates, Pors ***3***–***5***

In a 25 mL round-bottom flask, a mixture of Por **1** (100 mg, 8.3 × 10^−5^ mol) and α-(105 mg, 1.1 × 10^−4^ mol), β-(123 mg, 1.1 × 10^−4^ mol), or γ-cyclodextrin (140 mg, 1.1 × 10^−4^ mol) was dissolved in 5 mL of DMF. The reaction was carried out for 72 h at 60 °C under an N_2_ atmosphere, allowing for the nucleophilic substitution, also confirmed by TLC. The solvent was removed under reduced pressure and the reactional crude was dissolved in MeOH, then precipitated with water, filtered, and washed with cool water. The obtained purple solids were dried under a vacuum system over 6 h at 80 °C. The desired porphyrin–cyclodextrin conjugates were obtained in moderate yields (Por **3**: 47%, Por **4**: 58%, and Por **5**: 41%).

5-[4-(α-cyclodextrin)-2,3,5,6-tetrafluoro]-10,15,20-tris[2,3,5,6-tetrafluoro-4-(4-oxopyridin-1(4*H*)-yl)phenyl]porphyrin, Por **3**: ^1^H NMR (300.13 MHz, DMSO–*d*_6_): *δ* −3.08 (br s, 2H, -NH), 6.28–6.54 (m, 6H, *m*-H), 7.96–8.30 (m, 6H, *o*-H), 9.14–9.65 (m, 8H, β-H) ppm. ^19^F NMR (282.38 MHz, DMSO–*d*_6_): *δ* −145.98–−144.86 (m, 8F, Ar-F), −135.00–−133.69 (m, 8F, Ar-F) ppm. ESI–MS *m/z*: 1076.3 [M+2H]^2+^ and 710.9 [M+3H]^3+^. UV–Vis (DMSO): λ (log ε) 417 (4.17); 510 (2.01); 583 (1.47) nm.

5-[4-(β-cyclodextrin)-2,3,5,6-tetrafluoro]-10,15,20-tris[2,3,5,6-tetrafluoro-4-(4-oxopyridin-1(4*H*)-yl)phenyl]porphyrin, Por **4**: ^1^H NMR (300.13 MHz, DMSO–*d*_6_): *δ* −3.03 (br s, 2H, -NH), 6.27–6.76 (m, 6H, *m*-H), 7.92–8.20 (m, 6H, *o*-H), 9.22–9.43 (m, 8H, β-H) ppm. ^19^F NMR (282.38 MHz, DMSO–*d*_6_): *δ* −146.31–−145.17 (m, 8F, Ar-F), −135.57–−134.55 (m, 8F, Ar-F) ppm. ESI–MS *m/z*: 1157.5 [M+2H]^2+^ and 773.3 [M+3H]^3+^. UV–Vis (DMSO): λ (log ε) 417 (3.59); 509 (2.14); 584 (1.83) nm.

5-[4-(γ-cyclodextrin)-2,3,5,6-tetrafluoro]-10,15,20-tris[2,3,5,6-tetrafluoro-4-(4-oxopyridin-1(4*H*)-yl)phenyl]porphyrin, Por **5**: ^1^H NMR (300.13 MHz, DMSO–*d*_6_): *δ* −3.09 (br s, 2H, -NH), 6.39–6.66 (m, 6H, *m*-H), 7.83–8.40 (m, 6H, *o*-H), 9.17–9.75 (m, 8H, β-H) ppm. ^19^F NMR (282.38 MHz, DMSO–*d*_6_): *δ* −146.92–−144.68 (m, 8F, Ar-F), −135.72–−133.87 (m, 8F, Ar-F) ppm. ESI–MS *m/z*: 1239.5 [M+2H]^2+^ and 827.0 [M+3H]^3+^. UV–Vis (DMSO): λ (log ε) 418 (3.96); 510 (2.03); 584 (1.56) nm.

### 2.4. Synthesis of Cationic Por–CD Conjugates, Pors ***3a***–***5a***

In a sealed tube, Por **3** (50 mg, 2.3 × 10^−5^ mol), Por **4** (50 mg, 2.2 × 10^−5^ mol), or Por **5** (50 mg, 2.0 × 10^−5^ mol) was dissolved in 5 mL of DMF and reacted with an excess of (CH_3_)_2_SO_4_ (3.0 mL). The reaction was stirred overnight at 80 °C. After this period, the reaction tube was cooled to room temperature and the reactional mixture was precipitated with CH_2_Cl_2_ (6 mL), filtered, and washed with the same solvent. The obtained purple solids were dried under a vacuum system over 6 h at 80 °C and the cationic derivatives were obtained in moderate-to-good yields (Por **3a** (35 mg, 1.6 × 10^−5^ mol, 43%), Por **4a** (38 mg, 1.6 × 10^−5^ mol, 73%), Por **5a** (35 mg, 1.4 × 10^−5^ mol, 70%)).

5-[4-(α-cyclodextrin)-2,3,5,6-tetrafluorophenyl]-10,15,20-tris[2,3,5,6-tetrafluoro-4-(4-methoxypyridinium-1-yl)phenyl]porphyrin, Por **3a**: ^1^H NMR (300.13 MHz, DMSO-*d*_6_): *δ* −3.08 (br s, 2H, -NH), 4.37 (s, 9H, -OCH_3_), 8.14–8.20 (m, 6H, *m*-H), 9.37–9.55 (m, 14H, 6 *o*-H and 8 β-H) ppm. ^19^F NMR (282.38 MHz, DMSO-*d*_6_): *δ* −144.35–−143.47 (m, 8F, Ar-F), −133.88–−133.23 (m, 8F, Ar-F) ppm. UV–Vis (DMSO): λ (log ε) 416 (4.04); 509 (1.84); 583 (1.35) nm.

5-[4-(β-cyclodextrin)-2,3,5,6-tetrafluorophenyl]-10,15,20-tris[2,3,5,6-tetrafluoro-4-(4-methoxypyridinium-1-yl)phenyl]porphyrin, Por **4a**: ^1^H NMR (300.13 MHz, DMSO–*d*_6_): *δ* −3.08 (br s, 2H, -NH), 4.36 (s, 9H, -OCH_3_), 8.06–8.20 (m, 6H, *m*-H), 9.41–9.48 (m, 14H, 6 *o*-H and 8 β-H) ppm. ^19^F NMR (282.38 MHz, DMSO–*d*_6_): *δ* −144.32–−143.38 (m, 8F, Ar-F), −133.95–−133.42 (m, 8F, Ar-F) ppm. UV–Vis (DMSO): λ (log ε) 417 (3.37); 509 (2.14); 583 (1.61) nm.

5-[4-(γ-cyclodextrin)-2,3,5,6-tetrafluorophenyl]-10,15,20-tris[2,3,5,6-tetrafluoro-4-(4-methoxypyridinium-1-yl)phenyl]porphyrin, Por **5a**: ^1^H NMR (300.13 MHz, DMSO–*d*_6_): *δ* −3.10 (br s, 2H, -NH), 4.37 (s, 9H, -OCH_3_), 8.18–8.22 (m, 6H, *m*-H), 9.34–9.53 (m, 14H, *o*-H and β-H) ppm. ^19^F NMR (282.38 MHz, DMSO–*d*_6_): *δ* −144.08–−143.48 (m, 8F, Ar-F), −133.83–−133.27 (m, 8F, Ar-F) ppm. UV–Vis (DMSO): λ (log ε) 417 (3.10); 510 (1.82); 583 (1.43) nm.

### 2.5. Synthesis of Por–CD Conjugate, Por ***7***

Por **2** [17] (100 mg, 8.1 × 10^−5^ mol) and 10 equivalents of β-cyclodextrin were dissolved in 5 mL of DMF and the reaction was stirred under an N_2_ atmosphere and over 72 h at 60 °C until the confirmation, by TLC, that Por **2** was totally consumed. After the workup, the reactional residue was dissolved in MeOH and precipitated in water, filtered, and washed with water. The obtained purple solid constituted by Por **7** was dried under a vacuum system over 6 h at 80 °C. Compound **7** was obtained in a moderate yield (99 mg, 4.2 × 10^−5^ mol, 52%).

5-[4-(β-cyclodextrin)-2,3,5,6-tetrafluorophenyl]-10,15,20-tris[2,3,5,6-tetrafluoro-4-(pyridin-4-ylthio)phenyl]porphyrin, Por **7**: ^1^H NMR (300.13 MHz, DMSO–*d*_6_): *δ* −3.08 (br s, 2H, -NH), 7.39–7.96 (m, 6H, *m*-H), 8.40–8.63 (m, 6H, *o*-H), 9.37–9.60 (m, 8H, β-H) ppm. ^19^F NMR (282.38 MHz, DMSO–*d*_6_): *δ* −133.65 (dd, *J* = 26.4, 11.2 Hz, 8F, Ar-F), −128.70 (dd, *J* = 26.4, 11.2 Hz, 8F, Ar-F) ppm. ESI–MS *m/z*: 1187.2 [M+2H]^2+^. UV–Vis (DMSO): λ (log ε) 420 (3.13); 510 (2.00); 588 (1.65) nm.

### 2.6. Synthesis of Cationic Por–CD Conjugate, Por ***7a***

In a sealed tube, Por **7** (50.1 mg, 2.1 × 10^−5^ mol) was dissolved in DMF (3.0 mL) and was added to an excess of methyl iodide (1.0 mL, 16.1 mmol). The reaction was stirred overnight at 40 °C. After this period, the reactional mixture was cooled until room temperature and the positive charged product was precipitated in CH_2_Cl_2_, filtered, and washed with the same solvent. The obtained purple solid was dried under a vacuum system over 6 h at 80 °C and was recovered in excellent yield, obtaining the compound **7a** (48 mg, 1.9 × 10^−5^ mol, 95%).

5-[4-(β-cyclodextrin)-2,3,5,6-tetrafluorophenyl]-10,15,20-tris[2,3,5,6-tetrafluoro-4-(1-methylpyridinium-4-yl-thio)phenyl]porphyrin triodide, Por **7a**: ^1^H NMR (300.13 MHz, DMSO–*d*_6_): *δ* −3.08 (br s, 2H, -NH), 4.35 (s, 9H, *N*-CH_3_), 8.44–8.48 (m, 6H, *m*-H), 8.90–9.00 (m, 6H, *o*-H), 9.59–9.71 (m, 8H, β-H) ppm. ^19^F NMR (282.38 MHz, DMSO–*d*_6_): *δ* −132.89–−132.76 (m, 8F, Ar-F), −128.00–−127.87 (m, 8F, Ar-F) ppm. UV–Vis (DMSO): λ (log ε) 420 (3.09); 514 (1.95); 588 (1.62) nm.

### 2.7. Photostability Assays

The PBS solutions of Pors **3a**–**8a** (3 mL in quartz cuvettes) were irradiated under the same light conditions applied in the biological experiments. The UV–Vis spectrum was monitored, and the Soret band absorbance value (between 416 and 420 nm) of each PS (**3a**–**8a**) was displayed before (time *t*_0_ = *Abs*_0_) and after white light exposure for pre-defined intervals (5, 10, 15, 20, 25, 30, 45, and 60 min) (*Abs_t_*):

$$Photostability\;\left(\%\right)=\left(\frac{Abs_{t}}{Abs_{0}}\right)\times 100$$

#### Light Conditions

A white light LED system (ELMARK-VEGA20, 20 W, 1400 lm, range of 400–700 nm) was used for the irradiation of the Por dyes at different irradiation intensities of 25 and 50 mW cm^−2^. The light irradiance was determined through a Power Meter Coherent FieldMaxII196 Top with a Coherent PowerSens PS19Q energy sensor.

### 2.8. Singlet Oxygen Generation

Different solutions of each PS (Abs at 420 nm ≈ 0.2) were prepared in DMF with 9,10-dimethylanthracene (9,10-DMA) at a concentration of 50 µM. These solutions were irradiated in quartz cuvettes with blue light (λ = 420 nm). A solution of **TPP** in DMF was used as a reference (Φ_Δ_ = 0.65) [44,45]. The photooxidation kinetics of 9,10-DMA was studied by decaying the absorption of 9,10-DMA absorbance at 378 nm, the photooxidation results of 9,10-DMA were photosensitized by PSs **3a**–**8a,** and the reference was registered in a first-order plot [46,47,48]. Three independent experiments were performed.

### 2.9. Bacterial Strain and Growth Conditions

The genetically transformed bioluminescent *E. coli* Top10 (by the luxCDABE genes of the marine bioluminescent bacterium *Allivibrio fischeri*) was grown in a Tryptic Soy Broth medium (TSA, Merck), enriched with 50 mg mL^−1^ of ampicillin (Amp) and 34 mg mL^−1^ of chloramphenicol (Cm). Before the assay, one colony was placed into a flask with Tryptic Soy Broth (TSB, Merck), enriched with Amp and Cm, and was grown at 25 °C overnight under shaking at 120 rpm. Then, an aliquot was transferred into 10 mL of TSB under the same growth conditions until the stationary growth phase was achieved. An optical density of 600 nm (OD_600_) of 1.6 ± 0.1 corresponded to 10^8^ colony forming units per millilitre (CFU mL^−1^). The correlation of CFU mL^−1^ and the bioluminescence signal (in relative light units, RLUs) of the bioluminescent *E. coli* is described in the literature [49].

### 2.10. Photodynamic Inactivation Studies

The bacteria of *E. coli* were grown overnight in TBS. After that, it was diluted in PBS (pH = 7.49) to a final concentration of ≈ 10^7^ CFU mL^−1^, corresponding to ~10^7^ RLU. This bacterial suspension was equivalently distributed in 6-well plates and a suitable volume of each PS was added in order to accomplish the final concentration of 5.0 µM. From our previous published binding studies [17], the best concentration to minimize the aggregation effect of the PSs was 5 µM. The experiment had a light control (LC), which contained only the bacterial suspension, as well as a dark control (DC), which included the bacterial suspension incubated with the PS at 5.0 µM and was protected from light. Both were exposed to the same irradiation conditions as the samples. The samples and the controls were protected from light using aluminum foil and were kept in the dark for 15 min to provide the ability of PS to bind to *E. coli*. At the end of the incubation process, all the samples were irradiated under agitation for 60 min at 20 °C. Aliquots of 1.0 mL were collected and the bacterial bioluminescence was measured in a luminometer (TD–20/20 Luminometer, Turner Designs, Inc., Madison, WI, United States). The detection limit of the equipment is ca. 2.3 log. Three independent experiments were performed.

In addition to measuring bioluminescence, aliquots of treated and control samples were taken from the first and last time points, serially diluted, and poured in triplicate onto a Tryptic Soy Agar (TSA) medium. The plates were incubated for 24 h at a controlled temperature of 37 °C, and the concentration of the viable cells was determined by counting the CFU. At least three independent experimental measurements were conducted with three replicates. The results are determined as the average of the three assays.

### 2.11. Statistical Analysis

At least three independent experiments, with three replicates per assay for each condition, were done. The statistical analysis was performed with GraphPadPrism 8. The Kolmogorov–Smironov test was used to check for normal distributions, and the homogeneity of variance was verified with the Brown–Forsythe test. ANOVA and Dunnet’s multiple comparison tests were applied to assess whether the samples were statistically significant.

## 3. Results and Discussion

### 3.1. Synthesis, Photophysical, and Photochemical Characterization of the Porphyrin Derivatives

**Figure 1:**
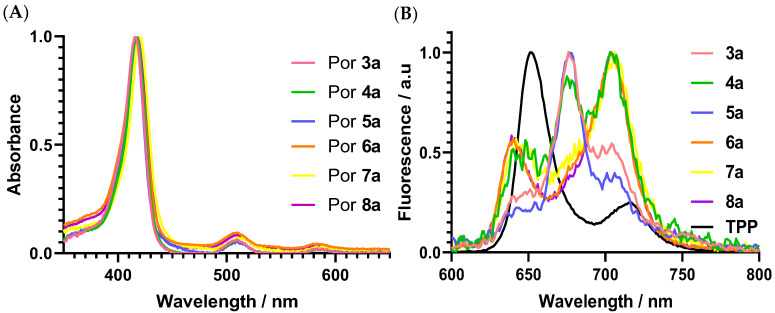
Normalized (**A**) absorption and (**B**) emission spectra of Pors **3a**–**8a** in DMSO at 298 K.

The neutral tri-substituted 4-pyridinone, Pors **1** and **3**–**5,** and the cationic Pors **3a**–**5a** (Scheme 1), were prepared through a nucleophilic substitution methodology well-established in our research group [50]. The tri-substituted 4-pyridinone Por **1** was prepared from commercial **H_2_TPPF_20_** using three equivalents of 4-hydroxypyridine and K_2_CO_3_ in DMF as the solvent. Pors **3**–**5** was synthesized from Por **1** after the reaction with ~1.2 equivalents of the corresponding α-, β-, or γ-cyclodextrin (Scheme 1), where the reactional temperature was carefully controlled. The cationic Pors **3a**–**5a** was attained by a quaternization process using an excess of dimethyl sulfate in DMF at 80 °C. It is worth noting that the quaternization of the pyridinone groups from **3**–**5** requires higher temperatures when compared with the quaternization of pyridyl groups **6**–**8** which occurs at 40 °C. Although the neutral Pors **3**–**5** were isolated with moderate yields (47%, 58%, and 41%, respectively), the cationic derivatives **3a**–**5a** were achieved in good yields (43%, 73%, and 70%, respectively). The substituted Pors were synthesized following experimental procedures established in our research group [17]. Por **2** (Scheme 1) was prepared from the **H_2_TPPF_20_** by the nucleophilic substitution with three equivalents of 4-mercaptopyridine in DMF. The neutral Pors **6**–**8** were synthetized from Por **2** with one α-, β-, or γ-cyclodextrin unit (Scheme 1) via nucleophilic substitution. The cationic Pors **6a**–**8a** were obtained from the cationization of Pors **6**–**8**, respectively, with methyl iodide in DMF. All Por structures were confirmed by ^1^H and ^19^F NMR spectroscopy, ESI–MS spectrometry (when it was possible to determine), and were characterized by UV–Vis absorption and emission spectroscopy (Figure 1).

The ^1^H NMR spectrum of the Por **1** is characteristic of a trisubstituted pattern, evidencing two doublets at *δ* 6.55 ppm and *δ* 8.23 ppm (*J* = 7.7 Hz) corresponding to the resonance of 6 *meta*-protons and 6 *ortho*-protons on the 4-pyridinone groups, respectively. Additionally, the ^19^F NMR spectrum of Por **1** is compatible with a *para* substitution pattern of three pentafluorophenyl rings and one unsubstituted pentafluorophenyl ring. The ^19^F NMR spectra of Pors **3**–**5** revealed the disappearance of the signal at −149.75 ppm due to the *para*-fluorine replacement by the cyclodextrin and the appearance of two multiplets on the interval of *δ* −146.92–−144.68 ppm and *δ* −135.72–−133.69 ppm, corresponding these signals to the *ortho*- and *meta*-fluorine atoms. The cationization of the neutral compounds provided to the Pors **3a**–**5a** were confirmed by the appearance of the singlet in the aliphatic region located at *δ* 4.36–4.37 ppm of the ^1^H NMR, which corresponds to the resonance of the -OCH_3_ protons.

The ESI–MS analysis shows the observed species resulting from the characteristic fragmentation processes of these Pors with the formation of species with different overall *m/z* ratios.

#### 3.1.1. Photophysical and Photochemical Studies

The absorption spectra of cationic Pors **3a**–**8a** were recorded in DMSO solutions (~10^−5^ M) at 298 K (Figure 1). The UV–Vis absorption spectra of the new Pors **3a**–**5a** and **7a** in DMSO solutions exhibit a typical feature of *meso*–substituted porphyrin derivatives with a strong Soret band at ca. 418 nm and weak Q bands between 500 and 650 nm (Figure 1). Moreover, although free-base porphyrins generally display four Q bands, *meso*-pentafluorophenylporphyrin derivatives often display only two Q bands, with the remaining two Q bands as indefinite. It is worth noting that the Soret bands of Pors **3a**–**5a** and **7a** appear slightly broadened in the base, typical of the aggregation phenomena. All the studied derivatives showed weak emissive properties with fluorescence quantum yields above 1% [6,50]. This fact might be due to a non-radiative excited deactivation that is very likely due to aggregation phenomena observed in DMSO for the tricationic dyes conjugated with a CD unit.

The absorption characteristics, molar extinction coefficients (ε), and fluorescence quantum yield for Pors **3a**–**8a** in DMSO are summarized in Table 1.

As previously mentioned, one of the key characteristics of a molecule that is considered a PS is its ability to generate singlet oxygen (^1^O_2_), one of the main ROS responsible for causing cell damage, leading to cell death. 

**Figure 2:**
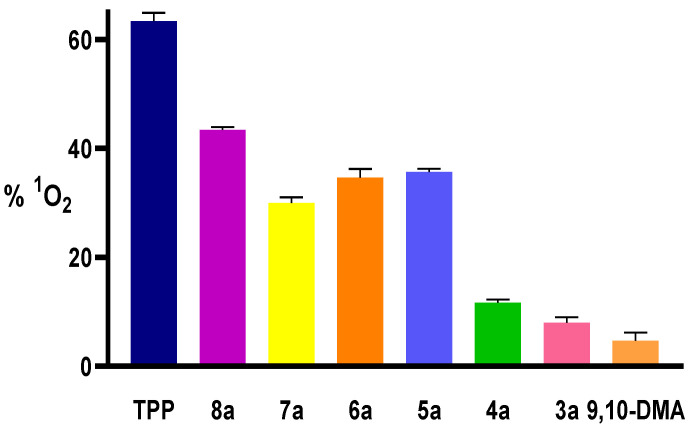
% ^1^O_2_ generation by Por–CD dyes **3a**–**8a** in DMF at 298 K.

Thus, the production of ^1^O_2_ by Pors **3a**–**8a** in DMF was evaluated by decaying the absorption of 9,10-DMA at 378 nm under light irradiation. No significant photodegradation of 9,10-DMA in DMF was observed without any PS under light irradiation. The generation of ^1^O_2_ by the new cationic Pors (**3a**–**5a** and **7a**) was compared to the known cationic Pors **6a**, **8a**, and a **TPP** reference (Φ_Δ_ = 0.65 in DMF) that is considered a good generator of ^1^O_2_ [44,45]. According to the obtained results summarised in Figure 2, all derivatives can generate ^1^O_2_ after light exposure. The gamma-cyclodextrin Por derivatives **5a** and **8a** are the best producers of ^1^O_2_ among the three cyclodextrins (α-, β-, and γ-derivatives) assessed. Comparing methoxypyridinium Por–CD dyes (**3a**–**5a**) with the corresponding thiopyridinium Por–CD dyes (**6a**–**8a**) [17], the results point out that thiopyridinium porphyrin derivatives display a higher ability to generate ^1^O_2_ species.

Since the application purpose is the use of Por–CD derivatives **3a**–**8a** under light conditions to photoinactive *E. coli*, their photostability was studied by monitoring the absorption decay of the Soret band after irradiation with white light (400–800 nm) at an irradiance of 50 mW cm^−2^ for 60 min. The obtained results are summarized in Table 2.

The Por–CD conjugates **3a**–**8a** showed excellent photostability in PBS solution, showing a robust behavior facing the irradiation used, with 86% to 94% of the porphyrin conjugates remaining unaltered after irradiation with white light at an irradiance of 50 mW cm^−2^ for 60 min (Table 2). It is worth noting that the Soret absorbance of these compounds in the dark decays ca. 3%. These absorption decreases might be related to a slight aggregation phenomenon that occurred in PBS.

#### 3.1.2. Photodynamic Inactivation of *Escherichia coli*

**Figure 3:**
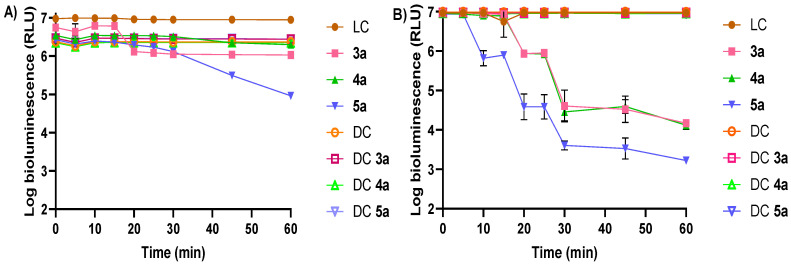
Bioluminescence monitoring of *E.* *coli* treated with PSs Por **3a**–**5a** at 5.0 µM at different irradiation times, under white light irradiation at (**A**) 25.0 mW cm^−2^ and (**B**) 50.0 mW cm^−2^. The values are expressed as the means of three independent experiments; error bars indicate the standard deviation; DC—dark control; LC—light control. Lines combine experimental points.

**Figure 4:**
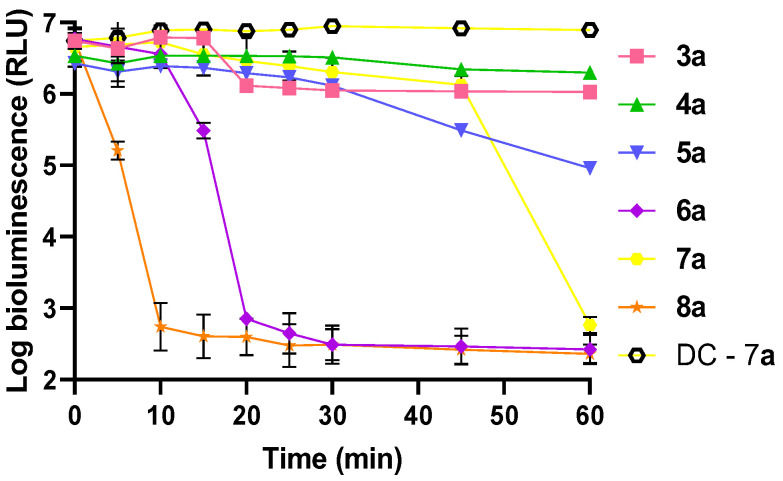
Comparison of photoinactivation of bioluminescent *E. coli* treated with methoxypyridinium Pors **3a**–**5a** and thiopyridinium Pors **6a**–8a* at 5.0 µM at different irradiation times, under white light irradiation at 25.0 mW cm^−2^. Values are expressed as means of three independent experiments; error bars indicate standard deviation. * The results for Pors **6a** and **8a** were described in the article [17].

The photodynamic efficiency of the cationic derivatives Por–CD was assessed against a bioluminescent *E. coli* strain Top10. The PDI efficiency of Pors **3a**–**5a** against the bioluminescent *E. coli* was evaluated at 5.0 μM under white light exposure at an irradiance of 25 mW cm^−2^ (Figure 3A) and 50 mW cm^−2^ (Figure 3B). The photodynamic efficiency of all Pors (**3a**–**8a**) (methoxypyridinium (**3a**–**5a**) and thiopyridinium (**6a**–**8a**)) was compared for the conditions of 5.0 μM and 25 mW cm^−2^ (Figure 4). As observed in Figure 3, no bacterial decay under the light conditions occurred in the absence of PS (data provided by the light control, LC) and the presence of the PS in the absence of light (data provided by the dark control, DC). It should be noted that the *E. coli* viability was not significatively affected by the cationic inverted Pors **3a**–**5a** at the light exposure at an irradiance of 25 mW cm^−2^ (60 min, Figure 3A). However, Por **5a** caused a slight *E. coli* photoinactivation, with a bioluminescence signal decrease of ~1.5 log_10_ (ANOVA, *p* < 0.0001). Nevertheless, when the light irradiance used was increased twice (50 mW cm^−2^, Figure 3B), the Por **5a** achieved a better PDI effect against the bioluminescent *E. coli*, with a ~3.5 log_10_ and 4.0 log_10_ reduction (ANOVA, *p* < 0.0001) after 30 and 60 min of light exposure, respectively, followed by Pors **4a** and **3a** (~2.5 log_10_ and ~3.0 log_10_, ANOVA, *p* < 0.0001) for the same light exposition time. The results point out the importance of the ^1^O_2_ generation by the PS. The photodynamic efficiency, to photoinactivate *E. coli,* of each PS **3a**–**5a** (Figure 3B) correlates with the ^1^O_2_ generation (Figure 2), in which the ^1^O_2_ production follows the ascending efficiency order (**3a** < **4a** < **5a**).

Comparing inverted methoxypyridinium Pors **3a**–**5a** and thiopyridinium Pors **6a**–**8a** at a concentration of 5.0 μM and an irradiance of 25 mW cm^−2^ (Figure 4), it was observed that the *E. coli* photoinactivation occurs faster mainly in the presence of Pors **6a** and **8a**. Once again, this highlights the importance of the ^1^O_2_ generation by the PS, reaching the detection limit of the methodology between 30–60 min of irradiation [17]. Pors **3a**–**5a,** at an irradiance of 50 mW cm^−2^, were less effective in the photoinactivation of the bioluminescent *E. coli* strain than the Pors **6a**–**8a**, even at an irradiance of 25 mW cm^−2^. This fact can be explained by the positive charge localization in the thiopyridinium conjugates (**6a**–**8a**) in an external position. Additionally, the cationic substituent type/CD unit and affinity to the outer bacterial structures cannot be ruled out. Overall, within each series of quaternized dyes (methoxypyridinium vs. thiopyridinium), the best PSs candidates were the cationic Por–γ–CD dyes **5a** and **8a** at irradiances of 50 mW cm^−2^ and 25 mW cm^−2^, respectively.

**Figure 5:**
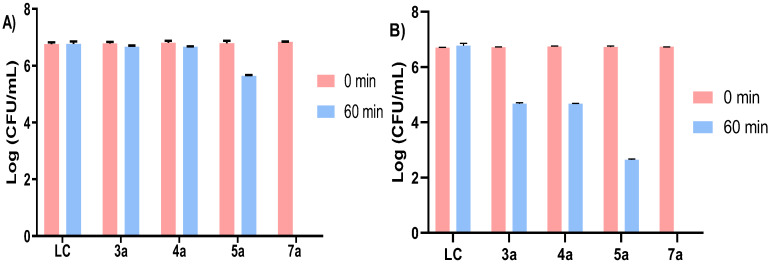
Photodynamic inactivation of bioluminescent *E. coli* treated with PSs **3a**–**5a** and **7a** at 5.0 µM after 60 min of white light irradiation at an irradiance of (**A**) 25 mW cm^−2^ and (**B**) 50 mW cm^−2^. The values are expressed as the means of three independent experiments; error bars indicate the standard deviation.

For a better consistency in our study, it was related to the reduction in *E. coli* bioluminescence with the reduction expressed in log_10_ CFU mL^−1^ accomplished with the pour-plate methodology at times 0 and 60 min. The obtained results are presented as the average of the three assays in Figure 5. The obtained results for the Por–CD derivatives **3a**–**5a** confirmed the results obtained through the bioluminescence monitoring of the PDI treatment. Only the conjugate **5a** was able to generate a decrease of ~1.5 log (ANOVA, *p* < 0.0001) in the viability of *E. coli* after 60 min of light irradiation. Besides, at the same irradiance (25 mW cm^−2^) and light exposure time (60 min), the Por–CD **7a** caused a total photoinactivation of the *E. coli*, which corroborated the determined bioluminescence results, and is similar to the PDI results found in the literature for Por–CD **6a** and **8a**^17^). When the irradiance was increased to 50 mW cm^−2^, Pors **3a**–**5a** caused a decrease of ~2 log_10_ to ~4 log_10_ in the viability of *E. coli*, and Por **7a** reached a complete bacteria inactivation. These results are in agreement with the RLU results found for photoinactivation bioluminescent *E. coli*. All obtained results confirmed that the external positive charge and type of the cyclodextrin substituent of the Por–CD dyes considerably influences the PDI efficiency.

It is well-known that the PS efficacy on the PDI treatments is correlated with different aspects, such as the microbial adsorption behavior, the ability of PS to generate singlet oxygen, the photostability profile, the solubility/aggregation comportment, or cell localization, among others [51]. Despite the fact that the ability to generate ^1^O_2_ for all cationic Por derivatives, the γ-cyclodextrin derivatives (**5a** and **8a**) were revealed to be better ^1^O_2_ generators. This fact might justify the more efficient *E. coli* inactivation profile achieved for Por–CD conjugates **5a** (3.5 log_10_ reduction, 60 min, 50 mW cm^−2^) and **8a** (4.0 log_10_ reduction, reaching the detection limit of the methodology, 15 min, 25 mW cm^−2^), and it was more pronounced for **8a** due to its highest ^1^O_2_ generation, good water solubility, and the maximized electrostatic interactions between their more exposed peripheral positive charges and the Gram-negative bacterium. Moreover, different solubility profiles in the physiological medium of Por–CD derivatives can also justify the distinct PS inactivation behavior of *E. coli*. It is widely known that γ-cyclodextrin is more water-soluble than α-cyclodextrin [51,52], which clarifies the fact that Por derivatives **5a** and **8a** (γ-cyclodextrin derivatives) tend to have better PDI performances compared with the others Por–CD conjugates.

It is also important to highlight that the structural manner in which hydroxypyridine reacts with the Por structure (**3a**–**5a**) can also influence the photoinactivaion efficiency. The substitution by the nitrogen, instead of the expected oxygen-bridge, as in a thio–bridge (**6a**–**8a**)**,** probably makes the former less flexible. In addition, the position of the positive charge may also contribute to the aggregation behaviour in water once it is more protected from the aqueous environment. The position of the positive charge plays a key role in the physicochemical and biological features of the Por conjugates, evidencing that these features should be further addressed in the pursuit of optimized PS for antimicrobial photodynamic inactivation.

## 4. Conclusions

The new free-base Pors **3**–**5**, **3a**–**5a**, **7,** and **7a** were prepared and structurally characterized, and the photodynamic efficiency of the cationic Por–CD conjugates **3a**–**5a** and **7a**, as well as of the known Pors **6a** and **8a**, were evaluated against the bioluminescent strain of *E. coli*. The antimicrobial PDI assays within the series of **3a**–**5a** (5.0 μM) showed that, only under white light at an irradiance of 50 mW cm^−2^, a significative *E. coli* photoinactivation occurred. The Por **5a** proved to be the best PS, causing a decrease of 3.5 and 4.0 log_10_ of the bioluminescence signal after 30 and 60 min of light irradiation, respectively. Analyzing the new inverted methoxypyridinium Pors **3a**–**5a** and the already-known thiopyridinium Pors **6a**–**8a**, at 5.0 μM at an irradiance of 25 mW cm^−2^, a faster (in 30 min) and complete *E. coli* photoinactivation was confirmed, particularly for thiopyridinium Pors **6a** and **8a**. The obtained results show that the methoxypyridinium Por–CD conjugates (**3a**–**5a**) evidence less effectivity against the bacterial viability than the thiopyridinium Por–CD conjugates (**6a**–**8a**). According to the American Society of Microbiology, this is higher than the minimum required (reduction > 3 log CFU mL^−1^) for a new approach to be termed as antimicrobial. The Por **5a** (50 mW cm^−2^) and Pors **6a**–**8a** (25 mW cm^−2^) can be considered promising PS drugs for PDI.

## Figures and Tables

**Table 1:** Absorption and emissive properties of Pors **3a**–**8a** in DMSO.

Pors	Soret Band (nm)	log ε	Q Bands (nm)	log ε	λ_emiss._	Φ_F_ *^a^*
**3a**	416	4.04	509	1.84	676	0.01
			583	1.35		
**4a**	417	3.37	509	2.14	704	<0.01
			583	1.61		
**5a**	417	3.10	510	1.82	677	<0.01
			583	1.43		
**6a** *^b^*	417	3.49	510	3.43	704	<0.01
			583	3.13		
**7a**	420	3.09	514	1.95	706	0.01
			588	1.62		
**8a** *^b^*	420	3.78	510	2.70	704	<0.01
			583	2.30		

*^a^* Using **TPP** (Φ_F_ = 0.13) as reference in DMSO [43]; *^b^* From reference [17].

**Table 2:** Photostability of Pors **3a**–**8a** in PBS after 60 min of white light irradiation at an irradiance of 50 mW cm^−2^.

Pors	3a	4a	5a	6a	7a	8a
**% Abs decay *^a^***	6	8	6	14	11	9

*^a^* Measured at Soret band wavelength.

## Data Availability

Not applicable.
